# Assessment of Dysphagia in Chinese Cohort of Angelman Syndrome: An Observational Study

**DOI:** 10.1111/cns.70587

**Published:** 2025-08-25

**Authors:** Yumeng Chen, Yanna Wang, Yi Zhang, Jun Wang, Xiaonan Du, Tianqi Wang, Yi Wang, Hao Zhou

**Affiliations:** ^1^ Department of Rehabilitation Children's Hospital of Fudan University, National Children's Medical Center Shanghai China; ^2^ Department of Clinical Epidemiology and Clinical Trial Unit Children's Hospital of Fudan University, National Children's Medical Center Shanghai China; ^3^ Department of Neurology Children's Hospital of Fudan University, National Children's Medical Center Shanghai China

**Keywords:** Angelman syndrome, dysphagia, gastrointestinal symptom, risk factor, swallowing function

## Abstract

**Aims:**

This study aims to identify the prevalence and risk factors of dysphagia in a Chinese cohort of Angelman syndrome (AS).

**Methods:**

A structured questionnaire was used to assess the status of patients in a Chinese cohort of AS. Swallowing function was evaluated using the Pediatric Eating Assessment Tool‐10, with gastrointestinal symptoms quantified via the Six‐item Gastrointestinal Severity Index (6‐GSI). To identify potential risk factors, univariable and multivariate logistic regression was performed.

**Results:**

Among 490 patients with AS (median 6 years, interquartile range 4 years), the molecular subtypes of 75.7% of cases were deletions of 15q11–q13. The prevalence of dysphagia reached 56.1%. Patients with dysphagia exhibited lower BMI values compared to nondysphagia cases (15.31 ± 2.87 vs. 15.92 ± 2.91 kg/m^2^, *p* = 0.021). Multivariate logistic regression analysis identified that uniparental paternal disomy (UPD) was associated with lower odds of dysphagia compared with deletions of 15q11–q13 (OR = 0.34, *p* = 0.016). Comorbid sleep disorders (OR = 1.79, *p* = 0.007), gastrointestinal disorders (OR = 1.89, *p* = 0.003), and increased 6‐GSI scores (OR = 1.16, *p* = 0.044) showed associations with higher odds of dysphagia.

**Conclusions:**

Over half of Chinese patients with AS experience dysphagia, with UPD moderating risk and comorbidities amplifying susceptibility.

## Graphical abstract

In Chinese cohort of Angelman syndrome (AS), the prevalence of dysphagia reached 56.1%, with uniparental paternal disomy associated with lower odds of dysphagia. Comorbid sleep disorders and gastrointestinal disorders, as well as higher scores on 6‐GSI, were linked to increased odds of dysphagia.

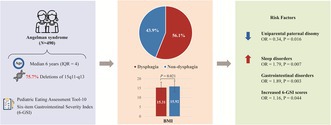

## 1 Introduction

Angelman syndrome (AS), a rare neurogenetic disorder first described in 1965 by Harry Angelman, has an estimated prevalence of 1:12,000–20,000 individuals [1, 2]. As comprehensively reviewed in the literature, this severe neurodevelopmental condition presents with characteristic clinical features including muscular hypotonia with hyperreflexia, ataxia, seizure disorders, microcephaly, and significant motor delays [3]. The pathogenesis of AS primarily involves functional impairment of the maternal ubiquitin‐protein ligase E3A (*UBE3A*) gene located at chromosomal locus 15q11.2–q13 [4]. Current molecular classifications delineate four distinct etiological mechanisms: (1) deletions of 15q11–q13 region, (2) *UBE3A* mutations, (3) uniparental paternal disomy (UPD), and (4) imprinting defects [5].

Neurogenetic developmental disorders comprise a genetically heterogeneous group of rare conditions characterized by early‐onset central nervous system developmental abnormalities. These disorders are frequently associated with complex multisystem comorbidities, including dystonia, motor dysfunction, epilepsy, autism spectrum disorder (ASD), and neuropsychiatric manifestations [6, 7]. Dysphagia and feeding difficulties emerge as core clinical features, particularly prevalent in AS, Prader–Willi syndrome, and Phelan‐McDermid syndrome [8, 9, 10]. Clinical recognition of swallowing dysfunction has increased in recent years, particularly within pediatric populations and neurological disorder cohorts [11, 12]. The World Health Organization's International Classification of Functioning, Disability and Health (ICF) formally defines swallowing function as “functions of clearing substances, such as food, drink and saliva through the oral cavity, pharynx and esophagus into the stomach at an appropriate rate and speed” [13]. Dysphagia, clinically defined as impaired swallowing efficiency and safety, arises from multifactorial etiologies. Primary causative factors include: (1) neurodevelopmental origins (e.g., preterm birth, neuromuscular pathologies), (2) structural anomalies of the aerodigestive tract, (3) gastrointestinal (GI) disorders, and (4) cardiopulmonary compromise [14].

Emerging research has highlighted dysphagia as a critical clinical concern in AS. Current clinical focus on swallowing dysfunction in patients with AS predominantly originates from its association with GI symptoms. From infancy, patients with AS demonstrate a high prevalence of feeding difficulties (66%; 95% CI 55%–75%), manifesting as diminished sucking reflex, impaired swallowing and frequent vomiting [10, 15]. Clinical study reveals gastroesophageal reflux and feeding‐related vomiting as predominant dysphagia manifestations, affecting 64% and 58% of pediatric AS cases respectively [16]. In patients with AS aged ≥ 16 years, gastroesophageal reflux prevalence remains elevated at 53% [17]. Dysphagia frequently persists into adulthood, with pneumonia incidence doubling in affected versus unaffected individuals [15]. Furthermore, previous study indicates differential dysphagia prevalence across molecular subtypes: 15q11‐q13 deletion carriers demonstrate higher incidence compared to UPD cases [18].

Dysphagia constitutes a major clinical challenge across the lifespan of individuals with AS. This persistent challenge underscores the necessity to establish precise epidemiological profiles and risk determinants, which are essential for developing evidence‐based multidisciplinary management strategies. Therefore, this study aims to identify the prevalence and risk factors of dysphagia in a Chinese cohort of AS.

## 2 Methods

### 2.1 Study Design and Participants

A structured questionnaire was developed to assess swallowing function and clinical characteristics in a Chinese AS cohort during a 7‐day data collection period (September 24–30, 2024). The questionnaire was developed by three rehabilitation specialists from the Rehabilitation Department at Children's Hospital of Fudan University, who worked together to reach a consensus on its contents.

Participants were recruited via the Angelman Syndrome Family Alliance (ASFA), a nationally recognized patient advocacy organization established in 2012. ASFA maintains China's largest AS registry (> 1500 families) and collaborates with multidisciplinary rare disease experts. ASFA advocates for “cultivating professional caregivers among parents” and addresses AS clinical challenges through comprehensive lifelong support, encompassing home‐based feeding guidance, rehabilitation protocols, epilepsy prevention/management, quality‐of‐life optimization, and caregiver psychological counseling. Concurrently, ASFA promotes translational research collaborations to advance therapeutic development.

### 2.2 Standard Protocol Approvals and Patient Consents

Ethical approval for this study was obtained from the Institutional Review Board of the Children's Hospital of Fudan University (Approval No. 2023[200]). Consent forms were obtained from legal guardians of all participants prior to study enrollment.

### 2.3 Data Collection

The web‐based questionnaire was developed and implemented through Wenjuanxing (www.wjx.cn), a secure online survey platform that generated unique QR‐coded access links. Trained personnel from the ASFA disseminated these digital identifiers to eligible families via their established communication networks. During the predefined 7‐day assessment window (September 24–30, 2024), caregivers accessed and completed the electronic survey by scanning the QR code using mobile devices.

The questionnaire comprised four domains:

1. Demographic characteristics: Documenting sex, height, weight, date of birth, and birthplace.
2. Clinical profiles: Recording diagnosis timeline, genetically confirmed molecular subtypes, and comorbid condition status.
3. Assessment of swallowing function.
4. Assessment of GI symptoms.

Additionally, questionnaires included retrospective data from deceased patients, capturing historical cases occurring prior to the study. Data collection for these cases included demographic characteristics, clinical profiles, date of death, and primary cause of death.

### 2.4 Assessment of Swallowing Function

The Pediatric Eating Assessment Tool‐10 (Pedi‐EAT‐10), a validated parent‐report instrument with established psychometric properties, was used for assessment of swallowing function. Originally developed in 2018 and cross‐culturally adapted into Chinese in 2024, this 10‐item instrument employs a 5‐point Likert scale (0 = no impairment; 4 = severe impairment) [19, 20]. A total score ≥ 4 indicates dysphagia.

### 2.5 Assessment of GI Symptoms

The Six‐item GI Severity Index (6‐GSI) was employed to evaluate the severity of GI symptoms. This validated instrument, originally developed in 2006 and subsequently modified in 2011, assesses six domains: constipation, diarrhea, stool consistency, stool smell, flatulence, and abdominal pain [21, 22]. Each domain is scored 0–2 (0 = asymptomatic; 2 = severe manifestation). Total scores demonstrate direct correlation with symptom severity, where elevated scores indicate greater GI symptom burden.

### 2.6 Statistics

Data analysis was conducted with IBM SPSS Statistics (Version 21.0). Continuous variables were reported as mean ± standard deviation (SD) or median with interquartile range (IQR). Group comparisons used Student's *t*‐test (normal distribution) or Mann–Whitney *U*‐test (non‐normal distribution), with normality assessed by Kolmogorov–Smirnov tests. Categorical variables were expressed as percentages and analyzed using chi‐square (*χ* ^2^) tests. Participants were stratified into dysphagia group (Pedi‐EAT‐10 ≥ 4) and nondysphagia group (Pedi‐EAT‐10 < 4) based on established cutoff criteria. Variables associated with dysphagia was identified by logistic regression analysis. Potential factors were first identified through univariate logistic regression. Variables demonstrating statistical significance (*p* < 0.05) or clinical relevance were subsequently included in the multivariable regression model. Results were presented using odds ratios (OR) accompanied by 95% confidence intervals (CI). A two‐tailed *p* value below 0.05 served as the threshold for determining statistical significance.

## 3 Results

### 3.1 General Cohort Characteristics

From 500 distributed questionnaires, 490 valid responses were retained for analysis (exclusion rate = 2%, due to participant mortality). Demographic characteristics of the cohort were presented in Table 1. Participants had a median age of 6 years (IQR = 4) and a mean BMI of 15.58 kg/m^2^ (SD = 2.90). The molecular subtype in 75.7% (371/490) of cases was deletions of 15q11–q13, while 68.2% (334/490) of participants were diagnosed before age 2 years. Epilepsy prevalence reached 81.0% (397/490), with 69.2% (339/490) experiencing seizure onset before age 3 years. Comorbidity analysis showed sleep disorders prevalence of 64.5% (316/490) and GI disorders prevalence of 46.3% (227/490). The median scores of Pedi‐EAT‐10 and 6‐GSI were 5 (IQR = 10) and 1 (IQR = 2), respectively.

**TABLE 1:** Demographic characteristics of the study population.

Characteristics	Total	Dysphagia group	Nondysphagia group	*p value*
Number	490	275 (56.1)	215 (43.9)	—
Pedi‐EAT‐10	5 (10)	10 (9)	0 (2)	—
Age (years)				
Median (interquartile range)	6 (4)	6 (4)	5 (4)	0.102
Range	0–22	0–22	0–21	
Sex [*n* (%)]				
Female	236 (48.2)	135 (27.6)	101 (20.6)	0.642
Male	254 (51.8)	140 (28.6)	114 (23.3)	
BMI (kg/m^2^)	15.58 ± 2.90	15.31 (2.87)	15.92 (2.91)	0.021*
Age of diagnosis [years, *n* (%)]				
0–1	91 (18.6)	57 (11.6)	34 (6.9)	0.568
1–2	243 (49.6)	133 (27.1)	110 (22.4)	
2–3	79 (16.1)	44 (9.0)	35 (7.1)	
> 3	77 (15.7)	41 (8.4)	36 (7.3)	
Molecular subtypes [*n* (%)]				
Deletions of 15q11–q13	371 (75.7)	218 (44.5)	153 (31.2)	0.007*
*UBE3A* mutations	75 (15.3)	43 (8.8)	32 (6.5)	
Uniparental paternal disomy	37 (7.6)	11 (2.2)	26 (5.3)	
Imprinting defects	5 (1.0)	3 (0.6)	2 (0.4)	
Not detected	2 (0.4)	0 (0)	2 (0.4)	
Epilepsy [*n* (%)]				
Yes	397 (81.0)	236 (48.2)	161 (32.9)	0.002*
No	93 (19.0)	39 (7.9)	54 (11.0)	
Age of first seizure onset (years)				
0–1	57 (11.6)	42 (8.6)	15 (3.0)	0.080
1–2	204 (41.6)	121 (24.7)	83 (16.9)	
2–3	78 (16.0)	41 (8.4)	37 (7.6)	
> 3	58 (11.8)	32 (6.5)	26 (5.3)	
Comorbidity [yes, *n* (%)]				
Scoliosis	78 (15.9)	59 (12.0)	19 (3.9)	< 0.001*
Sleep disorders	316 (64.5)	202 (41.2)	114 (23.3)	< 0.001*
GI disorders	227 (46.3)	159 (32.4)	68 (13.9)	< 0.001*
ASD	34 (6.9)	28 (5.7)	6 (1.2)	0.001*
ADHD	140 (28.6)	93 (19.0)	47 (9.6)	0.004*
Anxiety	24 (4.9)	22 (4.5)	2 (0.4)	< 0.001*
6‐GSI	1 (2)	1 (2)	1 (2)	0.002*

*Note:* Continuous variables are presented as mean ± standard deviation (SD) or median (interquartile range), and categorical variables are listed as *n* (%). One missing BMI value was replaced with the series mean calculated from available observations.

Abbreviations: 6‐GSI, six‐item GI severity index; ADHD, attention‐deficit/hyperactivity disorder; ASD, autism spectrum disorder; GI, gastrointestinal.

* *p* < 0.05.

### 3.2 Swallowing and GI Assessment Profiles

**FIGURE 1:**
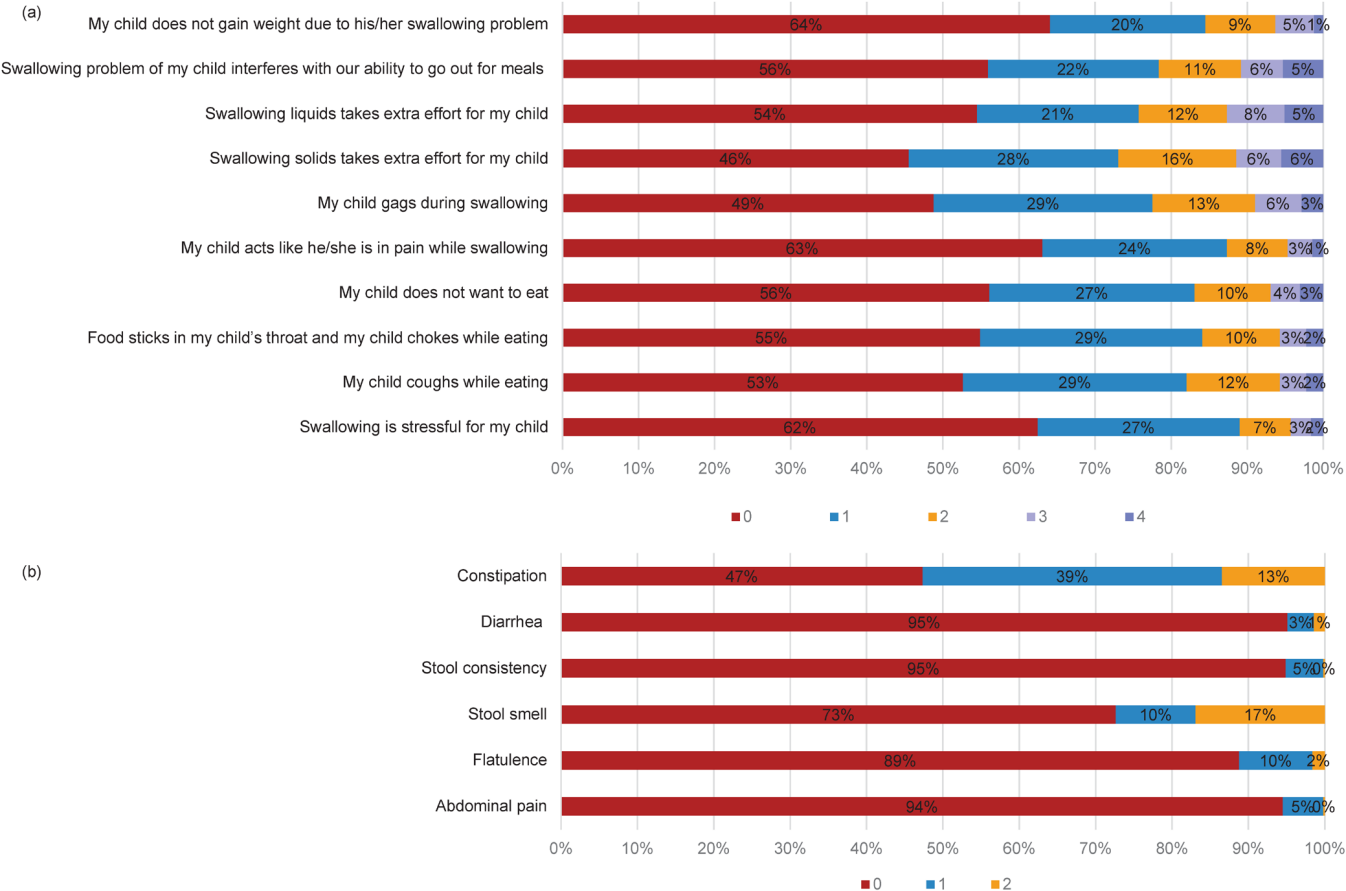
Comparative score distribution profiles. (a) Pediatric Eating Assessment Tool‐10 (Pedi‐EAT‐10): 10‐item instrument utilizing a 5‐point Likert scale (0 = no impairment; 4 = severe impairment). (b) Six‐item GI Severity Index (6‐GSI): Six gastrointestinal symptoms with 3‐level severity classification (0 = asymptomatic; 2 = severe manifestation).

Figure 1 (a) and (b) display the score distributions for individual items of the Pedi‐EAT‐10 and 6‐GSI, respectively. Scores of 0 indicate no clinically obvious symptoms, while scores from 1 to 4 reflect increasing symptom severity. The most prevalent swallowing‐related symptom, reported by 54% of the cohort, was increased effort during solid food ingestion. Conversely, impaired weight gain secondary to swallowing problems represented the least common symptom (36% prevalence). Among GI symptoms, constipation emerged as the most frequent complaint, affecting 53% of participants.

### 3.3 Clinical Comparisons Between Dysphagia and Nondysphagia Groups

Among 490 patients, those with Pedi‐EAT‐10 scores ≥ 4 (56.1%, 275/490) constituted the dysphagia group, while individuals scoring < 4 (43.9%, 215/490) served as controls. Table 1 presents comparative analyses of clinical characteristics and comorbidities in AS across groups. Cases and controls were similar in terms of age (*p* = 0.102), sex (27.6% female and 28.6% male vs. 20.6% female and 23.3% male, *p* = 0.642), and age of AS diagnosis (*p* = 0.568), but differed in terms of BMI (15.31 ± 2.87 vs. 15.92 ± 2.91 kg/m^2^, *p* = 0.021), molecular subtypes (*p* = 0.007), epilepsy (*p* = 0.002), comorbidities (scoliosis, *p* < 0.001; sleep disorders, *p* < 0.001; GI disorders, *p* < 0.001; ASD, *p* = 0.001; ADHD, *p* = 0.004; anxiety, *p* < 0.001) and 6‐GSI (*p* = 0.002).

### 3.4 Multivariable Logistic Regression Analysis

Univariable regression analysis (Table 2) identified associations between dysphagia risk and molecular subtypes (OR = 0.72, 95% CI 0.57–0.89, *p* = 0.003), epilepsy (OR = 2.03, 95% CI 1.28–3.21, *p* = 0.002), and multiple comorbidities. UPD was associated with lower risk of dysphagia compared to deletions of 15q11–q13 (OR = 0.30, 95% CI 0.14–0.62, *p* = 0.001); *UBE3A* mutations, imprinting defects, and undetected molecular defects were not different compared to deletions of 15q11–q13. Comorbid sleep disorders (OR = 2.45, 95% CI 1.68–3.58, *p* < 0.001), GI disorders (OR = 2.96, 95% CI 2.04–4.31, *p* < 0.001), and scoliosis (OR = 2.82, 95% CI 1.62–4.89, *p* < 0.001) exhibited strong associations. Neurodevelopmental and mental health conditions also showed notable correlations: ASD (OR = 3.95, 95% CI 1.60–9.72, *p* = 0.003), ADHD (OR = 1.83, 95% CI 1.21–2.75, *p* = 0.004), and anxiety (OR = 9.26, 95% CI 2.15–39.84, *p* = 0.003). Additionally, greater severity of GI symptoms measured by the 6‐GSI (OR = 1.23, 95% CI 1.09–1.40, *p* = 0.001) was associated with increased risk.

**TABLE 2:** Univariable and multivariate logistic regression analysis.

Variables	Univariable analysis		Multivariate analysis	
	OR (95% CI)	*p value*	OR (95% CI)	*p value*
Molecular subtypes	0.72 (0.57–0.89)	0.003*		0.100
Deletions of 15q11–q13	Reference		Reference	
*UBE3A* mutations	0.94 (0.57–1.56)	0.819	0.65 (0.47–1.59)	0.650
Uniparental paternal disomy	0.30 (0.14–0.62)	0.001*	0.34 (0.14–0.82)	0.016*
Imprinting defects	1.05 (0.17–6.38)	0.955	2.71 (0.40–18.23)	0.306
Epilepsy	2.03 (1.28–3.21)	0.002*	1.27 (0.70–2.32)	0.430
Comorbidity				
Scoliosis	2.82 (1.62–4.89)	< 0.001*	1.65 (0.90–3.02)	0.102
Sleep disorders	2.45 (1.68–3.58)	< 0.001*	1.79 (1.17–2.72)	0.007*
GI disorders	2.96 (2.04–4.31)	< 0.001*	1.89 (1.25–2.88)	0.003*
ASD	3.95 (1.60–9.72)	0.003*	1.88 (0.70–5.10)	0.212
ADHD	1.83 (1.21–2.75)	0.004*	1.26 (0.79–2.01)	0.342
Anxiety	9.26 (2.15–39.84)	0.003*	3.59 (0.77–16.73)	0.104
6‐GSI	1.23 (1.09–1.40)	0.001*	1.16 (1.00–1.34)	0.044*

Abbreviations: 6‐GSI, six‐item GI severity index; ADHD, attention‐deficit/hyperactivity disorder; ASD, autism spectrum disorder; GI, gastrointestinal.

* *p* < 0.05.

The multivariable logistic regression model (Table 2) identified associations between dysphagia risk and specific clinical variables. UPD was associated with a lower risk of dysphagia (OR = 0.34, 95% CI 0.14–0.82, *p* = 0.016) compared to deletions of 15q11–q13. Associations with increased dysphagia risk were observed for comorbid sleep disorders (OR = 1.79, 95% CI 1.17–2.72, *p* = 0.007), GI disorders (OR = 1.89, 95% CI 1.25–2.88, *p* = 0.003), and higher 6‐GSI scores (OR = 1.16, 95% CI 1.00–1.34, *p* = 0.044).

### 3.5 Characteristics of Mortality Cases

**FIGURE 2:**
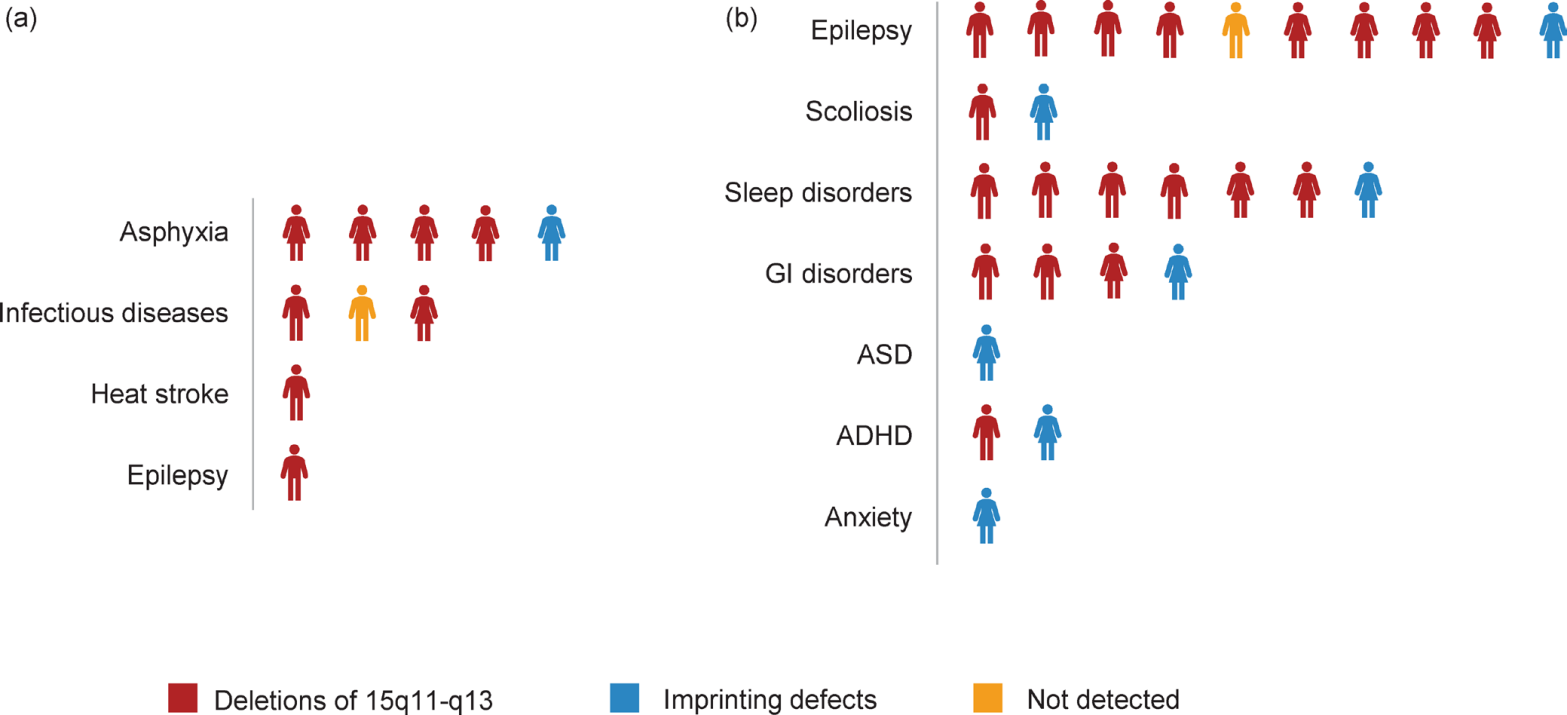
Characteristics of mortality cases. (a) Cause of death; (b) Premortem comorbidities. ADHD, attention‐deficit/hyperactivity disorder; ASD, autism spectrum disorder; GI, gastrointestinal.

A total of ten mortality cases were collected. As illustrated in Figure 2, asphyxia accounted for 50% of fatalities (*n* = 5), with four cases attributable to foreign body airway obstruction and one case resulting from sleep‐related positional asphyxia. The 15q11–q13 deletion subtype predominated among decedents (80%, *n* = 8). All mortality cases exhibited preexisting epilepsy, with one direct epilepsy‐related fatality.

## 4 Discussion

The present investigation revealed a 56.1% prevalence of dysphagia (275/490) in AS, with increased effort during solid food ingestion emerging as the principal clinical manifestation. These findings align with existing literature documenting feeding difficulties in 66% of infants, emesis during feeding in 58% of children and adolescents with AS, and dysphagia or frequent choking in 45% of adults with AS [10, 15, 16]. The marginally lower prevalence observed in our cohort likely reflects methodological distinctions, particularly the comprehensive age spectrum (0–22 years) and standardized assessment protocol employed. Notably, our study implemented the Pedi‐EAT‐10, a validated instrument demonstrating robust psychometric properties, contrasting with prior investigations reliant on unstructured symptomatic reporting. The congruence in dysphagia prevalence estimates across heterogeneous methodologies underscores the clinical significance of dysphagia as a core phenotypic feature in AS.

The current analysis demonstrated no associations between dysphagia risk and several comorbidities in AS, including epilepsy, ASD, ADHD, and anxiety. Epilepsy demonstrates high prevalence in AS, with individuals harboring 15q11–q13 deletions exhibiting younger seizure onset age and increased epilepsy severity [23]. In this cohort of 490 patients with AS, 81.0% (397/490) developed epilepsy, with dysphagia cases demonstrating a higher prevalence (48.2% vs. 32.9%, *p* = 0.002). Besides, 11.6% (57/490) experienced seizure onset within the first postnatal year, while 69.2% (339/490) manifested initial seizures by age 3 years, consistent with a previous study [24]. Although the pathophysiology linking dysphagia and epilepsy remains incompletely characterized, their co‐occurrence across neurodevelopmental disorders suggests potential shared mechanisms. Evidence indicates dysphagia may reduce medication compliance in epilepsy patients, potentially worsening seizure management and treatment complexity [25, 26, 27].

Molecular profiling identified deletions of 15q11–q13 as the predominant etiology in 75.7% (371/490) of the Chinese cohort of AS, followed by *UBE3A* mutations (15.3%, 75/490), UPD (7.5%, 37/490), imprinting defects (1.0%, 5/490), and undetermined genetic causes (0.4%, 2/490), consistent with established epidemiological patterns [5]. Multivariate logistic regression analysis revealed that UPD was associated with lower odds of dysphagia. In 2004, researches compared 49 deletion cases with 9 UPD cases, revealing a higher prevalence of hypotonia (73.3% vs. 28.6%) and swallowing disorders (73.9% vs. 22.2%) in the deletion group [18]. Consistent findings were observed in an AS mouse model, where *Ube3a*‐deficient mice displayed impaired swallowing coordination and altered licking rhythms associated with cerebellar dysfunction, with genotype‐specific behavioral variations underscoring the role of residual paternal *Ube3a* expression [28]. Furthermore, patients with UPD consistently demonstrated less severe clinical presentations, including developmental delays, seizures, mild microcephaly, and cognitive impairment, compared to patients with chromosomal deletion [18, 29, 30, 31, 32].

In this cohort study, 64.5% of patients exhibited comorbid sleep disorders, with higher prevalence in dysphagia patients (41.2%) compared to nondysphagia cases (23.3%). Logistic regression analysis further confirmed the association between sleep disorders and dysphagia risk. Previous research evaluated sleep disorders using the Sleep Disturbance Scale for Children in children and adolescents with AS, revealing a higher prevalence of sleep disorders at 85.45% [33]. A meta‐analysis revealed that sleep problems, characterized by frequent arousal episodes, somnolence, and possibly short sleep duration, represent the core sleep phenotype in AS [34]. Notably, it has been revealed that infant feeding history was not related to sleep disorders in children and adolescents with AS [33]. These findings underscore the necessity for longitudinal studies assessing swallowing function across developmental stages (infancy to adulthood) in patients with AS.

GI comorbidities and elevated 6‐GSI scores demonstrated associations with increased dysphagia risk in this cohort. Among 490 participants, 46.3% demonstrated comorbid GI disorders (32.4% in dysphagia vs. 13.9% in nondysphagia cases). Furthermore, patients with dysphagia showed higher 6‐GSI scores compared to nondysphagia cases (*p* = 0.002). Constipation emerged as the predominant GI symptom (53%), aligning with established literature. A study indicates that GI issues affect 87% of AS cases, with constipation (71%) and gastroesophageal reflux disease (GERD; 44%) representing the predominant manifestations [35]. In children and adolescents with AS, constipation was reported in 84% of patients, and gastroesophageal reflux was 64%, with gastroesophageal reflux predicting high‐frequency GI symptoms [16]. GERD serves as both a common etiological factor in esophageal dysphagia and a potential exacerbator of existing dysphagia manifestations [36, 37]. GERD and sleep disorders demonstrate a bidirectional relationship whereby nocturnal reflux symptoms impair sleep architecture through fragmentation and arousals, while sleep disturbances exacerbate GERD through visceral hypersensitivity, prolonged esophageal acid exposure, and ghrelin/leptin dysregulation [38, 39, 40].

## 5 Limitations

Focusing on the Chinese cohort of AS, this study evaluated the epidemiological characteristics of dysphagia, addressing critical evidence gaps in this understudied domain. The substantial sample size (*n* = 490) confers enhanced statistical power relative to prior studies, enabling robust characterization of dysphagia and associated clinical risk factors in AS populations. Furthermore, methodological rigor was strengthened through employment of the validated Pedi‐EAT‐10 for standardized swallowing evaluation. However, this study has three principal limitations. First, dependence on caregiver‐reported data introduces potential recall bias, underscoring the need for future investigations incorporating instrumental assessments like videofluoroscopic swallowing study (VFSS). Second, heterogeneous age distribution may mask developmental‐stage‐specific dysphagia patterns, necessitating age‐stratified longitudinal analyses to delineate ontogenetic trajectories. Finally, as an exploratory observational study, further research is essential to validate these findings and elucidate the underlying mechanisms.

## 6 Conclusion

Over half of patients experience dysphagia in the Chinese cohort of AS, characterized predominantly by increased effort during solid ingestion. UPD was associated with a lower risk of dysphagia, while coexisting sleep disorders or GI disorders heightened the risk. These findings highlight the clinical necessity of systematic dysphagia screening in patients with AS. Further research is essential to validate these associations and elucidate mechanisms underlying dysphagia in AS populations.

## Author Contributions

All authors contributed to this study, read and approved the final manuscript. Conceptualization: Yi Wang, Hao Zhou; Methodology: Hao Zhou, Yumeng Chen, Yanna Wang; Data analysis: Yumeng Chen, Yi Zhang, Yanna Wang; Data collection: Jun Wang, Xiaonan Du, Tianqi Wang; Writing – original draft preparation: Yumeng Chen, Yanna Wang; Writing – review and editing: Yi Zhang, Yi Wang, Hao Zhou; Funding acquisition: Yumeng Chen, Jun Wang, Yi Wang, Hao Zhou.

## Ethics Statement

Ethical approval for this study was obtained from the Institutional Review Board of the Children's Hospital of Fudan University (Approval No. 2023 [200]).

## Consent

Consent forms were obtained from legal guardians of all participants prior to study enrollment.

## Conflicts of Interest

The authors declare no conflicts of interest.

## Data Availability

To protect participant privacy, data not included in the publication may be shared in anonymized form upon request from qualified investigators seeking to replicate procedures and results, accessible through direct communication with the corresponding author.
